# Breast cancer and post-traumatic growth: A systemical review study

**DOI:** 10.1192/j.eurpsy.2021.1732

**Published:** 2021-08-13

**Authors:** C. Yastıbaş, İ.G. Yılmaz Karaman

**Affiliations:** 1 Psychology Department, Dokuz Eylül University, İzmir, Turkey; 2 Psychiatry Department, Eskişehir Osmangazi University, Faculty of Medicine, Eskişehir, Turkey

**Keywords:** post-traumatic growth, personality characteristics, breast cancer, coping

## Abstract

**Introduction:**

Breast cancer is a serious threat to people’s health. In addition to negative psychological disorders including depression, anxiety, and post-traumatic stress symptoms, positive changes such as post-traumatic growth (PTG) can be experienced.

**Objectives:**

The aim of this systematic review was to determine the variables related to PTG in people with breast cancer.

**Methods:**

We searched five database (SCOPUS, Cochrane, Medline, Science Direct, and Pubmed) starting from 1990, by guidance of PRISMA criteria, using the keywords “breast cancer”, “post traumatic growth”, “stress related growth”, and “benefit finding”.

**Results:**

There were conflicting findings regarding the relationship between PTG and following variables: sociodemographic variables such as age, education level, marital status, disease-related variables such as cancer stage, time since diagnosis, type of treatment. We observed that these variables may have a low effect on PTG. In addition, personality characteristics such as optimism, spirituality, and hope were found to be associated with PTG. Functional or problem-focused coping such as positive restructuring, acceptance, and religious coping, and ruminative thoughts predict PTG as a part of cognitive processing. Besides, social support has an important role in experiencing PTG.

**Conclusions:**

Psychosocial interventions for cancer patients are increasing day by day, but the scarcity of interventions which aims increase PTG is noteworthy. With this review, we recommend developing intervention programs that include functional coping strategies such as stress management, social skills training, cognitive techniques focused on ruminative thoughts, and positive restructuring.

**Disclosure:**

No significant relationships.

